# Is a motivational interviewing based lifestyle intervention for obese pregnant women across Europe implemented as planned? Process evaluation of the DALI study

**DOI:** 10.1186/s12884-017-1471-9

**Published:** 2017-09-07

**Authors:** Judith G. M. Jelsma, David Simmons, Nina Gobat, Stephen Rollnick, Kinga Blumska, Goele Jans, Sander Galjaard, Gernot Desoye, Rosa Corcoy, Fabiola Juarez, Alexandra Kautzky-Willer, Jürgen Harreiter, Andre van Assche, Roland Devlieger, Dirk Timmerman, David Hill, Peter Damm, Elisabeth R. Mathiesen, Ewa Wender-Ożegowska, Agnieszka Zawiejska, Annunziata Lapolla, Maria G. Dalfrà, Stefano del Prato, Alessandra Bertolotto, Fidelma Dunne, Dorte M. Jensen, Liselotte Andersen, Frank J. Snoek, Mireille N. M. van Poppel

**Affiliations:** 1 Department of Public and Occupational Health and, Amsterdam Public Health research institute, VU University Medical Centre, Van der Boechorststraat 7, 1081BT Amsterdam, Amsterdam, The Netherlands; 2 Institute of Metabolic Science, Addenbrookes Hospital, Cambridge, England UK; 3 Macarthur Clinical School, University of Western Sydney, Campbelltown, NSW Australia; 4 School of Medicine, Cardiff University, Cardiff, Wales UK; 5 Medical Faculty I, Poznan University of Medical Sciences, Poznan, Poland; 6 KU Leuven Department of Development and Regeneration: Pregnancy, Fetus and Neonate Gynaecology and Obstetrics, University Hospitals Leuven, Leuven, Belgium; 7 Department of Obstetrics and Gynaecology Division of Obstetrics and Prenatal Medicine Erasmus MC, University Medical Centre Rotterdam, Rotterdam, The Netherlands; 8 Department of Obstetrics and Gynecology, Medizinische Universität Graz, Graz, Austria; 9 Institut de Recerca de L’Hospital de la Santa Creu i Sant Pau, Barcelona, Spain; 10 CIBER Bioengineering, Biomaterials and Nanotechnology, Instituto de Salud Carlos III, Madrid, Spain; 11 Medical University of Vienna, Vienna City, Austria; 12 Recherche en Santé Lawson SA, St. Gallen, Switzerland; 13 Center for Pregnant Women with Diabetes, Departments of Endocrinology and Obstetrics, Rigshospitalet, Faculty of Health Sciences, University of Copenhagen, Copenhagen, Denmark; 14 Universita Degli Studi di Padova, Padova, Italy; 15 Università di Pisa, Pisa, Italy; 16 National University of Ireland, Galway, Ireland; 17 Odense University Hospital, Odense, Denmark; 18 Department of Medical Psychology, Amsterdam Public Health research institute, VU University Medical Centre, Amsterdam, The Netherlands; 19 Department of Medical Psychology, Academic Medical Centre, Amsterdam, The Netherlands; 20 Institute of Sport Science, University of Graz, Graz, Austria

**Keywords:** Counselling, Fidelity, Dose, Motivational interviewing, Lifestyle behaviour, Process evaluation, Pregnancy

## Abstract

**Background:**

Process evaluation is an essential part of designing and assessing complex interventions. The vitamin D and lifestyle intervention study (DALI) study is testing different strategies to prevent development of gestational diabetes mellitus among European obese pregnant women with a body mass index ≥29 kg/m^2^. The intervention includes guidance on physical activity and/or healthy eating by a lifestyle coach trained in motivational interviewing (MI). The aim of this study was to assess the process elements: reach, dose delivered, fidelity and satisfaction and to investigate whether these process elements were associated with changes in gestational weight gain (GWG).

**Methods:**

Data on reach, dose delivered, fidelity, and satisfaction among 144 participants were collected. Weekly recruitment reports, notes from meetings, coach logs and evaluation questionnaires (*n* = 110) were consulted. Fidelity of eight (out of twelve) lifestyle coach practitioners was assessed by analysing audio recorded counselling sessions using the MI treatment integrity scale. Furthermore, associations between process elements and GWG were assessed with linear regression analyses.

**Results:**

A total of 20% of the possible study population (reach) was included in this analysis. On average 4.0 (of the intended 5) face-to-face sessions were delivered. Mean MI fidelity almost reached ‘expert opinion’ threshold for the global scores, but was below ‘beginning proficiency’ for the behavioural counts. High variability in quality of MI between practitioners was identified. Participants were highly satisfied with the intervention, the lifestyle coach and the intervention materials. No significant associations were found between process elements and GWG.

**Conclusion:**

Overall, the intervention was well delivered and received by the study population, but did not comply with all the principles of MI. Ensuring audio recording of lifestyle sessions throughout the study would facilitate provision of individualized feedback to improve MI skills. A larger sample size is needed to confirm the lack of association between process elements and GWG.

**Trial registration:**

ISRCTN registry: ISRCTN70595832; Registered 12 December 2011.

**Electronic supplementary material:**

The online version of this article (10.1186/s12884-017-1471-9) contains supplementary material, which is available to authorized users.

## Background

Obesity and excessive gestational weight gain (GWG) are both independent risk factors for adverse outcomes and complications of pregnancy, such as gestational diabetes mellitus (GDM) [1]. The Institute of Medicine (IOM) has developed guidelines for GWG based on the pre-pregnancy body mass index (BMI) [2]. To prevent women from exceeding these recommendations, prevention trials are needed [3]. A number of lifestyle studies have been designed to assist in limiting GWG by providing an intervention focused on healthy eating, physical activity or a combined approach, although so far no definitive answer in favour of any of these interventions has been found [4–6].

Motivational Interviewing (MI) has been shown to be effective in helping persons change their lifestyle behaviours, such as physical activity and diet across different target populations [7–12] and might be an effective counselling technique for professionals to deal with the difficult situations experienced by obese pregnant women [13] and to assist in limiting GWG [14]. MI is a “collaborative, goal-oriented style of communication with particular attention to the language of change. It is designed to strengthen personal motivation for and commitment to a specific goal by eliciting and exploring the person’s own reasons for change within an atmosphere of acceptance and compassion” (p.29) [15].

The vitamin D and Lifestyle Intervention (DALI) study aims to investigate across a number of European centres, how effective a behavioural lifestyle intervention is in the prevention of GDM. In these centres, pregnant women with a BMI of ≥29 kg/m^2^ receive guidance from a lifestyle coach trained in MI [16]. The pilot study found lowest GWG with a healthy eating (HE) intervention relative to a physical activity (PA) or combined (HE + PA) intervention [17]. While these results seem promising they need to be confirmed in a larger trial including a control group. Furthermore, intervention components are complex in nature, so it is important to evaluate process variables to indicate how a planned intervention was conducted, especially when different persons delivered an intervention across many countries, as is the case in our study.

Process evaluations allow researchers to provide insight into why an intervention has shown positive or negative results [18, 19]. Moreover, in behavioural intervention research it is of utmost importance to report treatment fidelity, which refers to the “methodological strategies used to monitor and enhance the reliability and validity of behavioural interventions” (p.443) [20]. Miller and Rollnick (2014) underscore the importance for assessing the quality of the intervention when delivering MI in order to determine the effect of the quality of MI in the intervention on the results of the study [21]. Another important component is the description of strategies used to acquire a higher treatment fidelity that could be valuable for clinical practice in the actual implementation process [20].

The primary objective of this process evaluation was to assess reach (including recruitment), dose delivered, fidelity and satisfaction of the DALI lifestyle pilot study. The secondary objective was to investigate whether these process elements could explain differential effects of the intervention on changes in GWG.

## Methods

There are several approaches for conducting complex process evaluation, with no single best way to design a process evaluation [22]. The current paper uses dimensions from the framework of Linnan and Steckler (2002) [18]. The original framework includes seven dimensions: context, reach, fidelity, dose delivered and received, implementation and recruitment. This has some overlap with the five dimensions of the reach, effectiveness, adoption, implementation, and maintenance (RE-AIM) evaluation framework [23]. In Table 1 an overview of the developed process evaluation plan is presented, with a specification of the research questions, complete and acceptable delivery and used process measure according to Saunders et al. (2005) [24].

### DALI study

A detailed description of the cross-national DALI study is provided elsewhere [16]. In short, pregnant women with a body mass index (BMI) ≥ 29 kg/m^2^ were recruited between January 2012 and March 2013. All women who agreed to participate in the DALI project underwent a 2 h 75 g oral glucose tolerance test (OGTT) before 20 weeks of gestation, whereby those with GDM according to the International Association of Diabetes in Pregnancy Study Group (IADPSG) criteria were excluded [25]. The pilot lifestyle study was conducted in eleven study centres across nine European countries (Austria (Vienna), Belgium (Leuven), Denmark (Odense and Copenhagen), Ireland (Galway), Italy (Padua and Pisa), Netherlands (Amsterdam), Poland (Poznan), Spain (Barcelona) and the United Kingdom (UK) (Cambridge)). Local medical ethical committees in these countries approved the study and all participants gave written informed consent.

**Table 1:** Process evaluation plan

Process evaluation question	Complete and acceptable delivery	Process measure
How many people of the target population took part in the project? (reach)	The intervention group is comparable to the study population	Weekly recruitment report kept by each research centre
What recruitment procedures were used to attract pregnant women? (recruitment)	Strategies on recruitment across multicentre research.	Notes from (telephone) meetings with research nurses, coaches, principal investigators throughout the project
How many participants received 5 face-to-face conversations with a personal lifestyle coach? (dose delivered)	All (100%) received 5 face-to-face conversations with a personal lifestyle coach	Coach logs recorded after a session
To what extend was face-to-face counselling delivered as planned by MI guidelines? (fidelity)	MI was applied to deliver the face-to-face conversations	Recorded conversations assessed using the Motivational Interviewing Treatment Integrity measure (MITI 3.1.1)
How many telephone booster sessions did the participants receive? (dose delivered)	0–4 telephone booster sessions were delivered	Coach logs recorded after a session
How satisfied were participants with the DALI intervention (components)? (dose received: satisfaction)	All participants were satisfied with the DALI intervention	Evaluation questionnaire at 35–37 weeks of gestation

#### DALI intervention

Women were randomly allocated to one of three intervention arms; either a healthy eating (HE) arm, a physical activity (PA) arm or a combined HE and PA (HE + PA) arm. Between entry and 35 weeks of gestation five face-to-face sessions were planned supplemented with four optional telephone coaching sessions with a personal lifestyle coach, based on the principles of MI. Tools such as a pedometer, a dynaband, a food diary, action goal cards and a manual with information on GDM, appropriate GWG, HE and/or PA were provided to the participants, depending on the intervention randomization arm.

#### Lifestyle coaches

Key competencies for the lifestyle coach were: being empathic (understanding another person’s experience, feeling and behaviour), curious (willingness to explore the person’s experience), client focused (primary focus to help another person) and collaborative (equal perception of client and coach). The coach should not be dominant or chaotic (should be able to structure a conversation). In total twelve lifestyle coaches delivered the intervention sessions. In all the individual sites one coach was appointed, except in one site where a coach left the project and was replaced. All coaches were female with various professional backgrounds; most were PhD students with a master’s degree in either nutrition or human movement science; one was working as a midwife, one as a registered dietitian and one person had a background in information technology. Three coaches were familiar with MI prior to the start of this study, either by completing a local MI course or by acquiring experience in an earlier intervention study.

#### Training of the lifestyle coaches

Prior to the start of the pilot study a first 2-day central training was offered to the coaches in Cambridge (UK), and led by experienced MI trainers. The first training included an introduction to MI and its key concepts, followed by role-play exercises and video recordings. These interactive components allowed coaches to play the role of study participant and lifestyle coach to experience the impact of MI. Furthermore, coaches practiced core skills and received individualised feedback on their performance. The training covered the eight stages in learning MI [26], whereby the following skills were taught: 1) spirit of MI; 2) OARS (open questions, affirmations, reflections, summaries): client centred counselling skills; 3) recognizing change talk (desire, ability, need, reason and commitment) and sustain talk; 4) eliciting and strengthening change talk (by the use of OARS); 5) rolling with resistance; 6) developing a change plan/making an action plan (activation, commitment, taking steps); 7) consolidating commitment; and 8) transition and blending with the DALI intervention. A second two day training was delivered a few months later to review the coaches’ MI competency, share experiences and receive feedback on a role-play. The DALI coaches received in total 32 h of MI training.

The training was held in English, and it was recommended to non-English practitioners to contact a local and native language speaking MI trainer from the international Motivational Interviewing Network of Trainers (MINT-network) (Virginia, US; http://www.motivationalinterviewing.org) for further feedback based on an actual conversation in their own language.

### Reach

Reach is defined as the proportion of participants included in the intervention [24]. The numbers of persons eligible for participation (based on inclusion and exclusion criteria) were recorded in a weekly recruitment sheet at each site. Reasons for (not) participating in the DALI project were recorded. Approaching and attracting participants for a research project can be challenging, especially in a cross-national research project. Therefore, in research meetings with research nurses, coaches and principal investigators, recruitment strategies were discussed and recorded.

### Dose delivered

Dose delivered is defined as the amount or number of intended components delivered by the practitioner [24]. The number of face-to-face counselling sessions and telephone booster calls that were delivered to the participants was assessed. The aim was to deliver all (100%) of the five face-to-face conversations, while the telephone booster sessions were optional for the participants. It was preferred to deliver four face-to-face sessions prior to the second measurement at 24–28 weeks of gestation and to deliver the final face-to-face session prior to the last measurement at 35–37 weeks of gestation. Coaches kept information regarding dose delivered on a personal digital assistance (PDA) and uploaded this to a central database. A paper version of the PDA was kept in case of technical problems.

### Fidelity

Fidelity is defined as the extent to which the intervention was implemented as intended [24]. To assess fidelity, practitioners were asked to audio record all sessions (with permission of the participant). Fidelity of MI was assessed using the Motivational Interviewing Treatment Integrity (MITI 3.1.1.) scale [27]. The aim was to code at least four sessions of different patients throughout the pilot study of each practitioner to provide a reliable competency score for each practitioner on the MITI [28]. Finally, to assess an overall MI fidelity score for the DALI intervention, the individual scores for each practitioner were weighted for the total number of participants counselled.

#### Motivational interviewing treatment integrity (MITI 3.1.1)

The MITI 3.1.1. is a behaviour coding system that measures the extent to which a practitioner uses MI skills in a particular session. This instrument is widely used to test MI fidelity and has good reliability and sensitivity [29–32]. The MITI 3.1.1. has two components: global scores and behaviour counts. Coders used two ‘passes’ through the audiotape. The first pass is used to assess the global scores and the second pass to assign a behaviour count. Global scores were rated on a five point Likert scale for the following five dimensions: Evocation, Collaboration, Autonomy/support, Direction and Empathy. Adding the Evocation, Collaboration, Autonomy/support dimensions forms the global ‘spirit’ score. Furthermore all utterances were assigned a behaviour count: open and closed questions, simple and complex reflections, giving information, MI adherent (e.g. advise with permission, affirming, emphasizing the client’s control, supporting) and MI non adherent (e.g. advice without permission, confronting, directing). It is possible that some utterances remain uncoded (e.g. off-topic talk, self-disclosure statements, facilitative statements, structure statements). The following summary scores of the behaviour counts were formed: Reflection to question ratio; Percent Open questions; Percent Complex reflections; Percent MI-adherent. ‘Beginning proficiency’ for motivational interviewing is met in the following conditions: global scores ≥3.5, reflection to question ratio in favour of reflection ≥1, percent open questions is ≥50%, percent complex reflection is ≥40% and percent MI-adherent is ≥90% [27]. In Table 2 a more detailed explanation and thresholds of the MITI scores are provided.

**Table 2:** Motivational Interviewing Treatment Integrity 3.1.1 score explanation and threshold [27]

	Explanation	Threshold
Global score		
Evocation	the extent to which the practitioner conveys an understanding that motivation for change and the ability to move toward that change, reside mostly within the client and therefore focuses efforts to elicit and expand it within the therapeutic interaction	3.5
Collaboration	the extent to which the practitioner behaves as if the interview is occurring between two equal partners, both of whom have knowledge that might be useful in the problem under consideration	3.5
Autonomy/Support	the extent to which the practitioner supports and actively fosters client perception of choice as opposed to attempting to control the client’s behaviour or choices	3.5
Direction	the degree to which a practitioner maintain appropriate focus on a specific target behaviour or concerns directly tied to it	3.5
Empathy	the extent to which the practitioner understands or makes an effort to grasp the client’s perspective and feelings	3.5
Spirit	Average of the Evocation, Collaboration, Autonomy/support dimensions	3.5
Behavioural count		
Reflection: Questions	dividing the total amount of reflections by the total amount of questions	1:1
% Open Questions	dividing the amount of open questions by the total amount of closed and open questions	50%
% Complex Reflections	dividing the amount of complex reflections by the total amount of simple and complex reflections	40%
% MI Adherent	dividing the amount of MI-adherent statements by the total amount of MI adherent and MI non adherent statements combined	90%

#### Rating procedure

MITI 3.1.1. requires a 20-min randomly selected sample and a clear target behaviour goal. Additional study requirements were that the audio session was delivered prior to the second measurement, making effect correlations possible. Session were chosen randomly from the available recorded sessions (see Table 5). Furthermore the 20-min segment was chosen randomly within each recording, although the segment should start at the beginning of one of the intervention messages (either the discussion of risk factors of GDM, or weight gain targets, or one of the healthy eating or one of the physical activity messages) so off topic talk in the beginning or end, such as discussing time and place for the next appointment was not included in the segment. If off topic talk still occurred during the session this remained uncoded, which is according to the MITI 3.1.1.

#### Coders

The lifestyle coaches delivered the lifestyle intervention in their own language. Therefore the sessions needed to be assessed by different coders proficient in these languages.

The conversations in Belgium, Ireland, The Netherlands and UK were assessed by one of the authors (JJ) and a coder not involved in the study (VM). Both received separately an extensive MITI 3.1.1 training (40 h). Experience with coding according to the MITI 3.1.1 was gained by a supervised coding of 18 samples both in Dutch and English before coding the actual study sample. Global scores of the study sample were discussed to reach consensus, if no consensus was reached the average of both was taken; for behavioural counts, the average of both counts was taken. Inter-rater reliability was assessed on the double coded study sample.

In Spain (PL), Denmark (HFK) and Italy (GP) members from the international MINT network experienced in coding with the MITI 3.1.1 assessed the fidelity of the sessions. Due to pragmatic reasons and costs no second coder was appointed. In Spain the conversations were evaluated according to the MITI 3.0 [33], because a translated Spanish manual for the MITI 3.0 was available. The MITI 3.0 differs from the MITI 3.1.1 in only minor textual revisions.

### Satisfaction

In the self-developed evaluation questionnaire (see Additional file 1) the overall participant satisfaction with DALI was measured on a 10 point Likert scale [very negative (1)-very positive (10)]. In addition, participants were asked to rate their satisfaction with the usefulness of the intervention materials (manual, pedometer, dynaband) used in the DALI study on a 10 point Likert scale [not useful at all (1) – very useful (10)]. A satisfaction score for the manual consisted of an averaged score for different parts of the manual (general information, GDM, weight management, healthy eating, physical activity), which was dependent on the intervention arm a woman was allocated to. Satisfaction about the lifestyle coach was assessed with the following six items: overall, overall knowledge, knowledge of the intervention, “helped you rather than told you what to do”, attitude, and ability to support. All these items were measured on a 10 point Likert scale [could do a lot better (1) – excellent (10)] and averaged to obtain an overall score. Furthermore, participants could rate both face-to-face and telephone calls on a 10 point Likert scale [not useful at all (1) – very useful (10)].

### Effectiveness

We examined the association between trial processes (fidelity, dose, and a composite score of both) and one of the primary outcomes GWG. This can help explain why the interventions did or did not work. To assess GWG, participants were weighed on a weighing scale (SECA 888; SECA 877) wearing no shoes and light clothes to the nearest 0.1 kg. GWG was calculated by subtracting the baseline weight (before 20 weeks) from the weight measured at the final measurement (35-37 weeks of gestation).

### Data analysis

Inter-rater reliability of the MITI 3.1.1 was assessed with intra-class correlation coefficients (ICC) for the behavioural scores and Krippendorf Alpha (KALPHA) [34] for the global scores over the study sample that was double coded (*n* = 20) [28]. The norm for good reliability is a KALPHA of 0.8 or higher [35]. ICCs were calculated using a two-way mixed model for absolute agreement. The following guidelines for interpretation of ICCs were used: below 0.40 = poor, 0.40–0.59 = fair, 0.60–0.74 = good, 0.75–1.00 = excellent [36].

Linear regression analyses were performed to explore association between process elements (independent variable) and GWG (dependent variable), correcting for baseline BMI and the total number of weeks between measurements. The following independent variables were considered: number of face to face (F2F) conversations, total number of conversations (F2F and phone/email combined), and a dichotomized implementation score for the process element fidelity, which was formed by adding all those MITI 3.1.1 criteria that fulfilled ‘beginning proficiency’, whereby a score of four or more out of seven elements was considered as ‘high’ and a score below four as ‘low’ (in Table 2 those with high MI competency are indicated with an asterisk). An overall score for the variable ‘DALI as intended’ was scored as ‘yes’ or ‘no’ fulfilling the criterion of: “all those who received counselling from a lifestyle coach who fulfilled ‘beginning proficiency’ in four out of seven elements and who received all five face-to-face sessions”. All those participants that lacked follow up data were removed from the sample. *P* < 0.05 was considered to be statistically significant. Analyses were conducted with software IBM Statistical Package for Social Sciences (SPSS) version 20.1.

## Results

### Reach

**Fig. 1:**
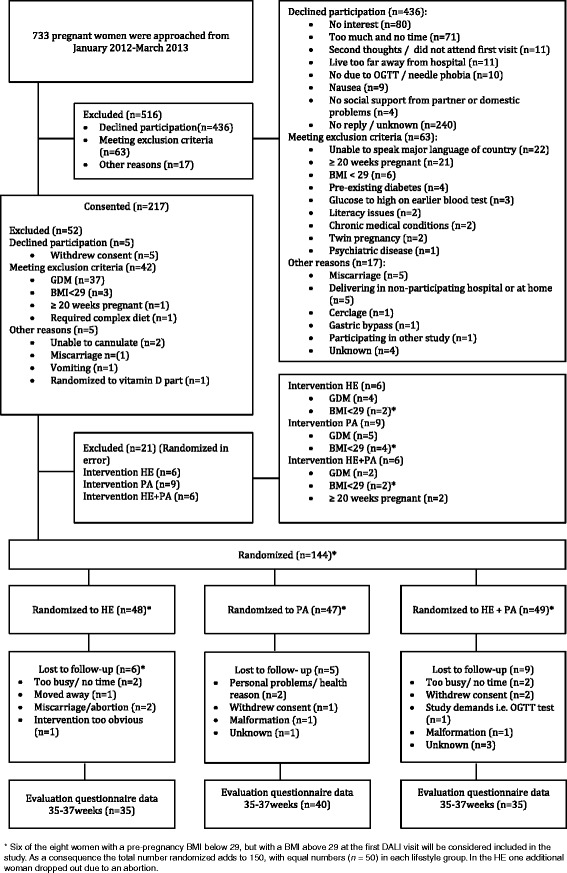
Flowchart of the DALI pilot study

In Fig. 1 the flowchart of this study is presented. A total of 144 participants from a total of 733 pregnant women who were invited (19.6%) were included in the DALI project. Of those that declined with a reason 41% reported not to be interested in this project and 36% thought it involved too much of their time to participate in this study. Of the 144 participants randomized to the DALI project, 48 were randomly allocated to the HE intervention, 47 to the PA intervention and 49 to the HE + PA intervention. A total of twenty participants dropped out during the study for various reasons. In one case this was due to the dislike of the HE intervention, the information provided was regarded ‘too obvious’.

Some deviations from the inclusion – exclusion criteria led to eight cases with a pre-pregnancy BMI lower than 29 who were randomized (in error) into one of the intervention-groups. Of these, six women had a BMI above 29 at the first DALI visit and are included in the DALI study and will be considered in future analysis. Still, one of these women dropped out of the HE intervention group due to a spontaneous abortion.

Each country applied different recruitment strategies based on differences in the organization of health care services (Table 3). Almost all countries actively invited women based on their BMI from their medical file or from referral from other parties such as obstetrician, midwife or general practitioner. In some cases the research nurse approached women in the waiting room of either the hospital or midwifery centre. Posters and leaflets about this ongoing study were presented, and two centres used TV advertisement in the waiting room. Ultrasound booking scans early in pregnancy were a perfect opportunity in some countries to invite women. In Italy all pregnant women received a booklet with information on all the currently ongoing studies. An advertisement on the hospital website and hosting a website in the local language made DALI visible online. Applied tactics related to pregnancy expositions, newspaper adds, involvement of leaflets in oversized clothes shops and child day care centres led to an extending visibility of DALI, although were less successful in actually recruiting participants. In Table 4, the recruitment numbers for each country are presented separately. The exclusion numbers for GDM were around 22%, although in some countries this was as low as 7% (UK), while in others as high as 44% (Denmark-Odense).

**Table 3:** Recruitment strategies applied across Europe

Strategies:	AUT	BEL	DNK- CO	DNK- OD	ESP	GBR	ITA	IRL	NLD	POL
Referral based on BMI:										
Medical file (hospital)	x	x	x	x		x		x	x	x
Early ultrasound	x	x			x	x	x			x
General practitioner			x	x					x	
Obstetrician	x	x			x	x	x	x	x	x
Midwife		x	x	x	x	x	x	x	x	
Community midwife					x	x			x	
Other clinics (e.g. antenatal clinic, endocrinology department, private obstetrician)	x				x		x	x		x
In medical setting:										
Approached by research nurse waiting room	x	x			x	x			x	
Information on all research projects combined							x			
TV advertisement in waiting room		x	x							
poster, leaflets advertisement	x	x	x		x	x	x	x	x	x
Hospital website advertisement					x					
Outside medical setting:										
Advertisement in newspaper					x			x	x	
Advertisement in clothe shops									x	
Advertisement in day-care									x	
Radio								x		
Pregnancy exhibition									x	
QR codes			x							
Website (local language)			x						x	

*Abbreviations*: *AUT* Austria, *BEL* Belgium, *DNK-CO* Denmark Copenhagen, *DNK-OD* Denmark Odense, *ITA* Italy, *IRL* Ireland, *NLD* The Netherlands, *ESP* Spain, *POL* Poland, *GBR* United Kingdom, *BMI* body mass index

**Table 4:** Recruitment numbers in each site in Europe

	AUT	BEL	DNK-CO	DNK-OD	ESP	GBR	IRL	ITA	NLD	POL	Total
Approached	91	67	84	140	39	118	46	45	71	32	733
Excluded	68	37	62	131	24	90	28	17	48	11	516
Declined	52	30	52	131	20	77	18	12	34	10	436
Excluded meeting exclusion criteria	15	6	5	0	3	9	7	5	13	0	63
Other reason	1	1	5	0	1	4	3	0	1	1	17
Consented	23	30	22	9	15	28	18	28	23	21	217
GDM	6	4	6	4	2	2	2	8	9	5	48
Other reason	1	3	2	2	2	8	5	1	1	0	25
Randomised	16	23	14	3	11	18	11	19	13	16	144

*Abbreviations*: *AUT* Austria, *BEL* Belgium, *DNK-CO* Denmark Copenhagen, *DNK-OD* Denmark Odense, *ITA* Italy, *IRL* Ireland, *NLD* The Netherlands, *ESP* Spain, *POL* Poland, *GBR* United Kingdom, *BMI* body mass index

The two most frequently mentioned reasons for participating in this study were the chance to reduce the risk of gestational diabetes (66%) and help society and science (55%). Learning more about lifestyle and pregnancy (36%) and receiving more ultrasound scans than usual (34%) helped subjects as well to decide to participate. Only 11% of the women indicated that more blood tests were a positive reason. A total of 30% of the women took part because someone (e.g. partner, friend, midwife, general practitioner) recommended this study.

### Dose delivered

On average 4.0 (SD ± 1.6) F2F conversations and 2.1 (SD ± 1.6) telephone conversations were delivered to the women. In total 63% of the participants received all five F2F sessions. Furthermore, 9% of the participants received a total of four sessions, 13% a total of three sessions, 3% a total of two sessions and 4% only one session. Directly after randomization 8% of the women dropped out and did not receive any lifestyle intervention. On average the first F2F conversation lasted 52 (SD ± 15) minutes, the second F2F conversation lasted 43 (SD ± 13) minutes, the third F2F conversation lasted 38 (SD ± 11) minutes, the fourth F2F conversation lasted 38 (SD ± 12) minutes and the final F2F conversation lasted 34 (SD ± 12) minutes. The duration of the telephone conversations was on average 14 (SD ± 8) minutes. A total of 42% of the participants received the intended four F2F sessions prior to the second measurement. Twelve Belgium women preferred email support instead of the optional telephone calls.

### MI Fidelity

No audio records from the pilot study were available from four coaches working in Austria, Denmark (Odense), Italy (Padua) and Poland. In Italy and Poland participants refused consent for recording of the conversations, which made the practitioners reluctant for asking each time. In Austria and Denmark, the practitioners were not aware of the required audio recording. The aim was to select at least four sessions of each practitioners, although due to fewer available recordings (#3, #5, #7) or the exclusion of some recorded session due to recorded time (#2) this was not obtained for certain practitioners. Practitioner #3 only had a session recorded on the behavioural counts. The analysed samples comprised 17.5% of the total available audio records. The results of each practitioner are presented in Table 5.

**Table 5:** Fidelity rating on the MITI variables

Overall scores based on n conversations	#1 (*n* = 4)	#2^a^ (*n* = 3)	#3^a,c^ (*n* = 1)	#4^a^ (*n* = 4)	#5 (*n* = 3)	#6^a^ (*n* = 8)	#7 (*n* = 2)	#8 (*n* = 3)	OVERALL	Inter rater reliability scores^b^
Global scores										*KALPHA*
Evocation (>3.5)	**3.8**	**3.7**	**-**	**3.8**	1.7	**4.4**	2.0	3.0	3.1	0.76
Collaboration (>3.5)	**3.5**	3.3	**-**	3.3	1.7	**4.3**	2.5	2.7	3.0	0.75
Autonomy / Support (>3.5)	**4.3**	**4.0**	**-**	**3.8**	2.3	**4.0**	**3.5**	3.0	**3.5**	0.52
Spirit (>3.5)	**3.6**	**3.7**	**-**	**3.6**	1.9	**4.2**	2.7	2.9	3.2	0.83
Direction (>3.5)	**5.0**	**5.0**	**-**	**4.0**	**3.7**	**4.9**	**4.0**	**5.0**	**4.5**	0.86
Empathy (>3.5)	3.3	**3.7**	**-**	**4.0**	2.7	**4.5**	2.0	3.0	3.3	0.86
Behavioural counts										*ICC*
GI	20.1	30.8	6.0	20.1	10.3	14.9	11.5	16.8	15.9	0.81
MIA	4.9	8.0	4.0	7.3	9.7	4.1	18.5	6.3	7.7	0.80
MINA	0.6	1.0	0.0	1.8	12.0	0.3	14.5	1.3	3.8	0.55
CQ	13.0	18.5	1.0	16.0	8.7	10.2	26.0	12.8	12.8	0.83
OQ	4.9	7.2	5.0	8.3	5.7	6.3	12.0	6.5	6.9	0.71
SR	6.3	13.5	8.0	6.4	2.3	10.1	19.5	7.3	9.0	0.48
CR	0.1	4.7	6.0	5.6	5.7	10.8	4.5	0.5	4.6	0.91
% Open Questions (>50%)	29	28	**83**	36	41	37	31	34	41	0.64
% Complex Reflections (>40%)	2	24	**43**	**44**	**68**	**52**	13	7	31	0.85
Reflections/Questions ratio (>1.0)	0.36	0.71	**2.33**	0.52	0.78	**1.45**	0.76	0.40	0.94	0.58
% MI Adherent (>90%)	87	**90**	**100**	81	52	**95**	49	85	81	0.46
Number of pilot participants	9	11	14	10	12	12	11	18	97	
N recorded conversations/N total conversations	18/47	10/51	1/65	41/42	3/53	44/53	2/44	41/62		

Bold values represent scores are above ‘beginning proficiency’ according to the MITI 3.1.1. Numbered columns refer to individual coaches

*Abbreviations*: *GI* giving information, *MIA* MI Adherent, *MINA* MI non Adherent, *CQ* closed question, *OQ* open question, *SR* simple reflection, *CR* complex reflection; ^a^Considered high in MI competence; ^b^Intraclass reliability scores based on 20 out of the samples of Belgium, Ireland, Netherlands and UK (total of 5 coaches); ^c^Only one session was recorded on the behavioural counts

The practitioners had varying levels of MI skilfulness. All coaches had a global Direction score according to ‘beginning proficiency’. Three practitioners (#2, #4, #6) reached a ‘beginning proficiency’ level for the global scores Spirit and Empathy. Two practitioners (#3 and #6) reached almost a ‘beginning proficiency’ level for all the behavioural counts as well, although most practitioners were far from these required levels. Two practitioners (#5 and #7) scored many MI non adherent statements (12 and 14.5 respectively). These statements were a result of advice giving without (implicit) permission. Overall, after correcting for the number of participants counselled in this study the MI scores reached ‘beginning proficiency’ for the global scores Direction and Autonomy/support, but scored below the cut off for the behavioural counts.

The inter-class reliability for the English and Dutch conversations on the behaviour counts ranged from fair to excellent (see Table 5). The global ratings Spirit, Empathy and Direction were all in the range for good reliability.

### Satisfaction

The overall DALI intervention received an 8.6 (SD ± 1.4) from the participants. The practitioners were rated with a 9.2 (SD ± 1.1). The F2F conversations were rated with an 8.8 (SD ± 1.5) and the phone calls with an 8.2 (SD ± 1.9). The women in the HE + PA group or in the PA group gave a 7.7 (SD ± 2.5) for the exercise dynabands and an 8.4 (SD ± 1.9) for the pedometer. A total of 16% rated the exercise dynabands and 6% the pedometer a 5 or lower (somewhat useful). The received manual was rated in the HE + PA group with an 8.2 (SD ± 0.2) in the HE group with an 8.4 (SD ± 0.3) and in the PA group with a 7.6 (SD ± 0.3). Only 9% of the women rated the manual with a 5 (somewhat useful) or lower.

### Effectiveness

Baseline and follow up GWG data was available for a total of 105 participants and was used in the analysis. Table 6 shows no significant associations between process elements and GWG. Although not statistically significant, in the PA group the participants who were counselled by a practitioner who reached ‘beginning proficiency’ in MI had 3.1 kg less GWG (95%CI: -7.0 to 0.8) compared to those who were counselled by a practitioner who did not reach ‘beginning proficiency’ in MI. In the HE group those who received more F2F sessions had 1.9 kg (95% CI: -0.8 to 4.6) more GWG, although this was also not statistically significant and partly caused by one outlier who lost 7 kg and attended only one lifestyle session.

**Table 6:** Association for dose and fidelity with change in gestational weight from first to last measurement across the three lifestyle groups

Process elements: dose, context and fidelity	HE	HE + PA	PA	Gestational weight gain (kg)	Gestational weight gain (kg)	Gestational weight gain (kg)
	Mean (SD) *N*	Mean (SD) *N*	Mean (SD) *N*	β (95%CI)	β (95%CI)	β (95%CI)
				HE	HE + PA	PA
Dose:						
Total number of F2F conversations	4.5 (1.0)	4.7 (0.6)	4.4 (1.2)	1.89	−0.89	−0.21
	34	34	37	(−0.78; 4.55)	(−3.45; 1.67)	(−1.60; 1.18)
Total number of contacts (F2F + phone/email)	7.5 (1.8)	7.1 (1.8)	6.6 (2.1)	0.08	−0.33	0.02
	34	34	37	(−1.41; 1.57)	(−1.23; 0.57)	(−0.75; 0.78)
Fidelity:						
Competence in Motivational Interviewing (high vs. low)^a^	0.50 (0.51)	0.54 (0.51)	0.42 (0.50)	1.11	−0.26	−3.13
	26	24	26	(−4.27; 6.49)	(−5.23; 4.71)	(−7.03; 0.77)
DALI as intended (MI + 5F2F) (yes vs. no)^b^	0.42 (0.50)	0.50 (0.51)	0.42 (0.50)	−0.32	−0.70	−3.13
	26	24	26	(−5.37; 5.09)	(−5.42; 4.02)	(−7.03; 0.77)

*F2F* face-to-face, *HE* healthy eating, *PA* physical activity, *HE + PA* healthy eating and physical activity, *MI* motivational interviewing. Gestational weight gain was calculated by subtracting the baseline weight from the weight measured at the final measurement and is corrected for BMI at baseline and total weeks between baseline and third measurement. If significant associations (*p* < 0.05) were found these were printed in bold. Significant negative beta’s regression coefficients represent a beneficial effect (decline in weight gain) and vice versa

^a^‘high’ corresponds with four or more out of seven MITI elements according to ‘beginning proficiency’ on the MITI

^b^‘yes’ corresponds with a lifestyle coach who was more competent in MI and a participant who received five face-to-face sessions

## Discussion

This process evaluation yields valuable information about the implementation process of the DALI lifestyle pilot study in overweight and obese pregnant women during the course of pregnancy. Delivering an intervention across different countries, with several different languages and various cultures is a real challenge. Evaluation is therefore important and could assist researchers and practitioners in planning future studies. This study reached a subset of the target population and eventually included 20% of the invited women. Overall, the intervention was delivered satisfactorily with 63% of the women receiving all intervention sessions. Furthermore, high variability between practitioners for MI competency resulted in an overall MI score not fulfilling expert opinion. Neither the implementation of MI (fidelity) nor the degree of participation in the intervention (dose delivered) was associated with the primary study outcome GWG.

With regard to reach, one-third of the invited women were willing to participate in this study, which is similar to the response rate of this target population in other intervention studies [37, 38]. The low response rate has implications for the generalizability of the results, as this sample was self-selective and research participants are mostly more motivated to change lifestyle behaviour in comparison to non-responders [39]. The requirement for women to attend three measurement sessions on top of the coaching sessions likely led to lower participation rates, as many women mentioned that the study demanded too much of their time. Additionally, ten women had a previous negative experience with the OGTT and would definitely not undergo this testing once more. On the other hand, the design of the study including individual F2F sessions with delivery at home (if preferred), might have resulted in a higher attractiveness and flexibility for women to participate than studies that require attending group sessions on fixed times and places. Furthermore, the incorporation of healthcare professionals ((community) midwifes, obstetricians, general practitioners) during the recruitment phase definitely led to an extended noticeability.

In comparison to previous studies the dose delivered of the F2F sessions is high, since for example in the UPBEAT pilot study only 6% attended the complete intervention (one F2F session followed by eight weekly group sessions) [37] and in the FitFor2 study only 16.3% attended half of the exercise sessions [38]. Similar attendance rates were found in the LIP study where 92% attended the four dietary counselling sessions and 56% attended half of the intended exercise sessions [40]. Only higher attendance rates were found in a study among Hispanic overweight women, in which 86% received all six prenatal lifestyle sessions [41].

The fidelity of the intervention delivery did not achieve scores above the MITI summary thresholds, although high variance of MI competence was observed between the practitioners. It is very likely that earlier experiences, professional background, personality and culture led to more (or less) skill quality and use of MI principles and as a consequence to a difference in the deliverance of the intervention across sites. This study was not designed to employ already highly skilled coaches. It was designed as a pragmatic trial to relate closely to the situation in actual clinical practice, whereby practitioners were trained in the beginning of this study under ideal training conditions, but needed to deliver their skills in the less predictable field setting. The delivery of this intervention was standardized (e.g. manuals for the coach, manuals for the participant, standardized training) to give practitioners some guidance, but obviously there is an inherent tension between a manualised intervention and the use of MI [42]. So far, the optimum amount of structure to offer practitioners is unclear when wanting them to integrate MI into practice. Certification of practitioners was based on attendance and completion of all requirements, not on quality in using MI skills. Future studies might reconsider this design and build audio recorded supervision into the training process, so coaches receive a mix of workshops and supervision (feedback) of actual practice over a longer time period, which would allow them to develop competence [21, 43–46]. A recent meta-analysis of training studies recommends 3–4 supervision sessions over a six-month period [45]. Supervisors should try to improve the quality of practitioners in evoking change talk (encouraging language about desire, ability, reason and need for behaviour change and commitment to it) and softening sustain talk (avoiding the focus on reasons against changing or maintaining the current situation) [47], since the expression of change talk is directly linked to the practitioners behaviour [48]. In addition, training practitioners in asking permission prior to giving advice is important in research studies were some form of knowledge transfer should harmonize with an MI approach.

The participants were highly satisfied with the intervention and with their lifestyle coach, even though this study was not able to reach a sufficient proficiency level in MI. One of the components in the coach rating was the item ‘helped you rather than told you what to do’, which is one of the key goals of MI. All participants scored an eight or higher for this item, with the exception of two participants (score of seven and a score of a three) both for a coach considered less skilled in the use of MI.

The secondary aim of this research paper was to investigate the association of dose and fidelity with change in GWG. In the previously published study of Simmons et al. (2015), it was shown that women gained 7.6, 8.5 and 9.6 kg respectively in the HE group, the HE + PA group and the PA group [17]. Purely hypothetical, the non-significant positive association found in the PA group for fidelity and GWG might suggest that the skilfulness of MI is essential when focusing on changing physical activity behaviours. This might suggest a need for training health professionals to a certain skill level of delivering behavioural change interventions, especially since gestational weight gain in the PA group was largest. However the sample size of the current study was probably the foremost reason no significant associations were found between dose, fidelity and GWG. Repeating this analysis in a larger study is needed to confirm the dose-response (more sessions better weight results) relation found in a non-pregnant population [49, 50].

### Strength and limitations

One has to balance in conducting a trial such as this between weighing up what is possible to achieve pragmatically and the potential loss of completeness in fidelity data. Not having double coding of the Spanish, Danish and Italian study session due to costs, the use of the Spanish MITI 3.0, and presenting only behavioural count data of #3 are limitations of this study, but the alternative was not to have this data, as was the case in previous MI studies among this target population [51, 52]. We believe these data are of great value for the evaluation process. A lesson learned from the pilot study is that throughout the study the collection of audio recordings should be ensured [28]. In the current study we lacked data from four practitioners, and of the other practitioners not all sessions were audio recorded, which might have led to a biased sample. Another limitation of this study is the gathering of satisfaction data at the end of pregnancy, therefore the response applied only to those who completed the study.

Notwithstanding the previous mentioned limitations, the findings of the present study are important. They provide insight in the ‘black box’ of the DALI pilot lifestyle intervention and allow for a more in-depth analysis of the outcomes. This study provides useful information across different countries, with its various health services, and is valuable for those planning to use MI in future intervention studies both in research and in practice. Moreover, it is one of the few studies that analysed fidelity of MI. Also, in contrast to other process evaluations, we investigated the association between separate process elements and the primary outcome measure of the study.

## Conclusion

Overall, these findings suggest that the DALI intervention with five MI lifestyle sessions was well received across a range of European countries and may even be feasible to implement more widely across Europe. Even though the performance of most of the practitioners in this study left room for improvement, some practitioners did adequately apply certain principles. Key lessons learned from this pilot study include; 1) that more research is needed to investigate which program adaptations are needed to attract the large group of non-responders to participate in a lifestyle intervention during pregnancy; 2) ensuring audio recording of all lifestyle sessions throughout the study; and 3) providing individualized feedback to practitioners throughout the study to increase the chances of achieving MI proficiency. Furthermore, future studies are encouraged to evaluate and report fidelity of MI in their study as to facilitate comparison among trials.

## Additional file

Additional file 1: Lifestyle process evaluation questionnaire. Process evaluation questions to gather reasons for participation and satisfaction with the DALI lifestyle study. (PDF 137 kb)

## Abbreviations

BMI: Body mass index
F2F: Face-to-face
GDM: Gestational diabetes mellitus
GWG: Gestational weight gain
ICC: Intra-class correlation coefficient
KALPHA: Krippendorff’s Alpha
MI: Motivational interviewing
MITI: Motivational Interviewing Treatment Integrity Scale
