# Supplementary material for: Is a motivational interviewing based lifestyle intervention for obese pregnant women across Europe implemented as planned? Process evaluation of the DALI study
Source: BMC Pregnancy Childbirth. 2017 Sep 7;17:293. doi: 10.1186/s12884-017-1471-9 (PMC5590191; doi:10.1186/s12884-017-1471-9)
Supplement: Additional file 1: — Lifestyle process evaluation questionnaire. Process evaluation questions to gather reasons for participation and satisfaction with the DALI lifestyle study. (PDF 137 kb) [file 12884_2017_1471_MOESM1_ESM.pdf]

LIFESTYLE EVALUATION QUESTIONNAIRE	
**Study ID number**	
**Date**	

EVALUATION QUESTIONNAIRE: Your chance for your say!

We would like to know something about your experience of being on the DALI study.

This questionnaire should be completed during your 35 to 37 weeks gestation visit. Please answer the following questions and then hand them back to the research midwife.

Section A. **General**

1. What was the reason for you to take part in the DALI study? ***Please tick all that apply***

   ☐ To help society/science learn more
   ☐ To learn more about lifestyle and pregnancy
   ☐ Someone (husband, friend, midwife, GP) recommended the study
   ☐ I wanted to reduce my chance of gestational diabetes
   ☐ To get more scans
   ☐ To get more blood tests
   ☐ Other – please specify

2. How do you rate your overall participation in the DALI study? ***Please circle***

   1 2 3 4 5 6 7 8 9 10

   Very negative Neutral Very positive

   Why did you rate DALI in this way?

3. What have you had to pay for by participating in the study? ***Please comment***

4. Please rate your satisfaction with the following points concerning your recruitment into the study. ***Please circle***

	Not at all satisfied				Moderately satisfied					Extremely satisfied
Way you were approached to participate	1	2	3	4	5	6	7	8	9	10
Communication with the research team	1	2	3	4	5	6	7	8	9	10
Participant information leaflet design	1	2	3	4	5	6	7	8	9	10
Participant information leaflet understandability	1	2	3	4	5	6	7	8	9	10
Participant information leaflet content	1	2	3	4	5	6	7	8	9	10
Consent form design	1	2	3	4	5	6	7	8	9	10
Consent form understandability	1	2	3	4	5	6	7	8	9	10
Consent form content	1	2	3	4	5	6	7	8	9	10

5. Please rate your satisfaction with the following points concerned with the study visits. ***Please circle***

	Not at all satisfied				Moderately satisfied					Extremely satisfied
Number of visits	1	2	3	4	5	6	7	8	9	10
Length of visits	1	2	3	4	5	6	7	8	9	10
Planning / organisation of the visits	1	2	3	4	5	6	7	8	9	10
Research team	1	2	3	4	5	6	7	8	9	10
Tests that were undertaken	1	2	3	4	5	6	7	8	9	10

6. Have your expectations about the study been met? Did it work out as you expected based on the information provided that you were given? ***Please circle***

Yes / No

If no, please give details

7. Do you have further suggestions or comments about your participation in the DALI study? ***Please comment***

Section B. **Lifestyle intervention**

1. How would you rate your coach?

	Could do a lot better				OK					Excellent
Overall	1	2	3	4	5	6	7	8	9	10
Overall Knowledge	1	2	3	4	5	6	7	8	9	10
Knowledge of the intervention	1	2	3	4	5	6	7	8	9	10
Use of the palm top computer	1	2	3	4	5	6	7	8	9	10
Helped you rather than told you what to do	1	2	3	4	5	6	7	8	9	10
Attitude	1	2	3	4	5	6	7	8	9	10
Ability to Support	1	2	3	4	5	6	7	8	9	10

2. How would you rate the face-to-face coaching sessions with your coach? **Please circle**

1 2 3 4 5 6 7 8 9 10

Not useful at all — Somewhat useful — Very useful

Any comments?

3. How would you rate the telephone conversations with your coach? **Please circle**

1 2 3 4 5 6 7 8 9 10

Not useful at all — Somewhat useful — Very useful

Any comments?

4. Rate the frequency of contact with the lifestyle coach **Please tick**

	Too many	Just right	Too few
The number of the face-to-face coaching sessions			
The number of telephone conversations with the coach			

Any comments?

5. How useful were the materials used? **Please circle**

	Not useful at all				Somewhat useful					Very useful
Manual, in general	1	2	3	4	5	6	7	8	9	10
Manual content about gestational diabetes	1	2	3	4	5	6	7	8	9	10
Manual content about weight gain management	1	2	3	4	5	6	7	8	9	10
Manual content about healthy eating	1	2	3	4	5	6	7	8	9	10
Manual content about physical activity	1	2	3	4	5	6	7	8	9	10
Glossary	1	2	3	4	5	6	7	8	9	10
Exercise Bands	1	2	3	4	5	6	7	8	9	10
Pedometer	1	2	3	4	5	6	7	8	9	10
Action Cards	1	2	3	4	5	6	7	8	9	10

6. Did you make any changes in your lifestyle? ***Please circle***

Yes / No

7. Will you maintain these lifestyle changes also after your pregnancy? ***Please circle***

Yes / No / Maybe

8. What were barriers for you to change your physical activity? ***Please tick all that apply***

    - Fatigue, feeling tired
    - Laziness
    - Lack of energy
    - Lack of motivation
    - Lack of enjoyment
    - Not feeling well (sick or ill)
    - Being anxious about risks and dangers
    - Other priorities (work, study, household, children)
    - Pregnancy complications (low back pain, nausea, constipation)
    - Physical limitations (injury, overweight)
    - Embarrassment about appearance
    - Lack of social support (partner, family, friends and/or co-workers)
    - Lack of time
    - Costs
    - Weather situation
    - Lack of appropriate assistance with child care
    - Lack of exercise facilities
    - Neighbourhood safety
    - Lack of information about physical activity
    - Lack of companionship for exercise
    - Other – please specify

9. What helped you change your physical activity? ***Please tick all that apply***

    - Motivation (to feel good, healthy and fit for myself)
    - Motivation (to take care of my baby)
    - Social support (partner, family, friends and /or co-workers)
    - Social support (coach, general practitioner, midwife)
    - Weather
    - Exercise classes, group program
    - Fixed schedules
    - Accessible childcare
    - Information about physical activity in general
    - Information about physical activity in relation to diabetes and health
    - Other – please specify

10. What were barriers for you to change your diet? ***Please tick all that apply***

- o Too expensive
- o Busy lifestyle
- o Lack of energy
- o Lack of motivation
- o Pregnancy complications (nausea, constipation)
- o Taste
- o Dislike of healthy foods by others in household
- o Lack of information about foods
- o Anxiety
- o Tiredness
- o Sickness
- o Cravings
- o Stress
- o Habits
- o Social occasions
- o Holidays
- o No social support (family, friends and/or co-workers)
- o Other – please specify

11. What helped you change your diet? ***Please tick all that apply***

- o Motivation (to feel good and healthy for myself)
- o Motivation (to take proper nutrients for my baby)
- o Social support (family, friends and /or co-workers)
- o Social support (coach, general practitioner, midwife, dietician)
- o Other – please specify

Section C. **Comments**

Please give details of any other feedback that you would like to give about any aspect of the study and your participation (please continue over if necessary).

Thank you for you for completing this questionnaire.
